# Oxic microbial ferrihydrite reduction rates of *Shewanella oneidensis* and the potential for Fe mobilization in oxic sediments

**DOI:** 10.1038/s41598-025-16963-w

**Published:** 2025-08-26

**Authors:** Giulia Ceriotti, Alice Bosco-Santos, Jasmine S. Berg

**Affiliations:** 1 Institute of Earth Surface Dynamics, Faculty of Geoscience and Environment, University of Lausanne, Lausanne, Switzerland; 2 Institute of Earth Sciences, Faculty of Geoscience and Environment, University of Lausanne, Lausanne, Switzerland

**Keywords:** Microbial iron reduction, Oxygen, *Shewanella oneidensis*, Anoxic microsites, Oxic sediments, Ferrihydrite, Element cycles, Water microbiology, Soil microbiology, Hydrology, Fluid dynamics

## Abstract

Microbially mediated reduction of ferrihydrite (Fe(III) oxyhydroxide) plays a crucial role in Fe cycling, and hence nutrient and contaminant cycling, in subsurface environments. This process is typically considered a strictly anaerobic process confined to anoxic microsites within oxic subsurface environments. However, recent findings suggest that microbes can also mediate ferrihydrite reduction under oxic conditions. Here, we quantified cell-specific rates of ferrihydrite reduction by the model organism *Shewanella oneidensis* MR-1 under oxic and anoxic conditions. We used these rates to assess the relative contribution of oxic and anoxic pore spaces to Fe(II) mobilization in a previously published laboratory analog of oxic aquifer sediments. Oxic reduction proceeded persistently, albeit at a per cell rate 100 times more slowly than anoxic reduction, yet still generated appreciable Fe(II). Modeling suggests that when anoxic microsites are absent or occupy a minor fraction of the pore space, oxic Fe(III) reduction can account for a significant share of total Fe(II) release. Such conditions are common in shallow aquifers, well-drained soils, and capillary fringes. We conclude that oxic Fe(III) reduction is a persistent background process that has been underestimated in current biogeochemical frameworks.

**Supplementary Information:**

The online version contains supplementary material available at 10.1038/s41598-025-16963-w.

## Introduction

The microbial reduction of ferric iron (Fe(III)) is a key anaerobic process driving natural subsurface biogeochemistry and releasing mobile ferrous iron (Fe(II)) to the environment^1^. The occurrence and the extent of microbial mobilization of Fe(II) depend on the ability of microorganisms to access and reduce solid Fe(III) minerals, the most reactive of which are considered to be amorphous oxides like ferrihydrite (Fe₂O₃·nH₂O). These minerals represent one of the main reservoirs of microbially accessible Fe(III) in soils and natural sediments.

Ferrihydrite exhibits a high and non-selective adsorptive capacity^2,3^ for contaminants such as arsenic and chromium, as well as nutrients like phosphate and nitrate^3–6^. Consequently, the spatial distribution of ferrihydrite and the extent of its reductive dissolution in soils and sediments are closely linked to the rate of organic carbon mineralization, the mobility of toxic compounds and micronutrients as Fe(II), and overall soil fertility in agricultural systems^7–9^.

**Fig. 1:**
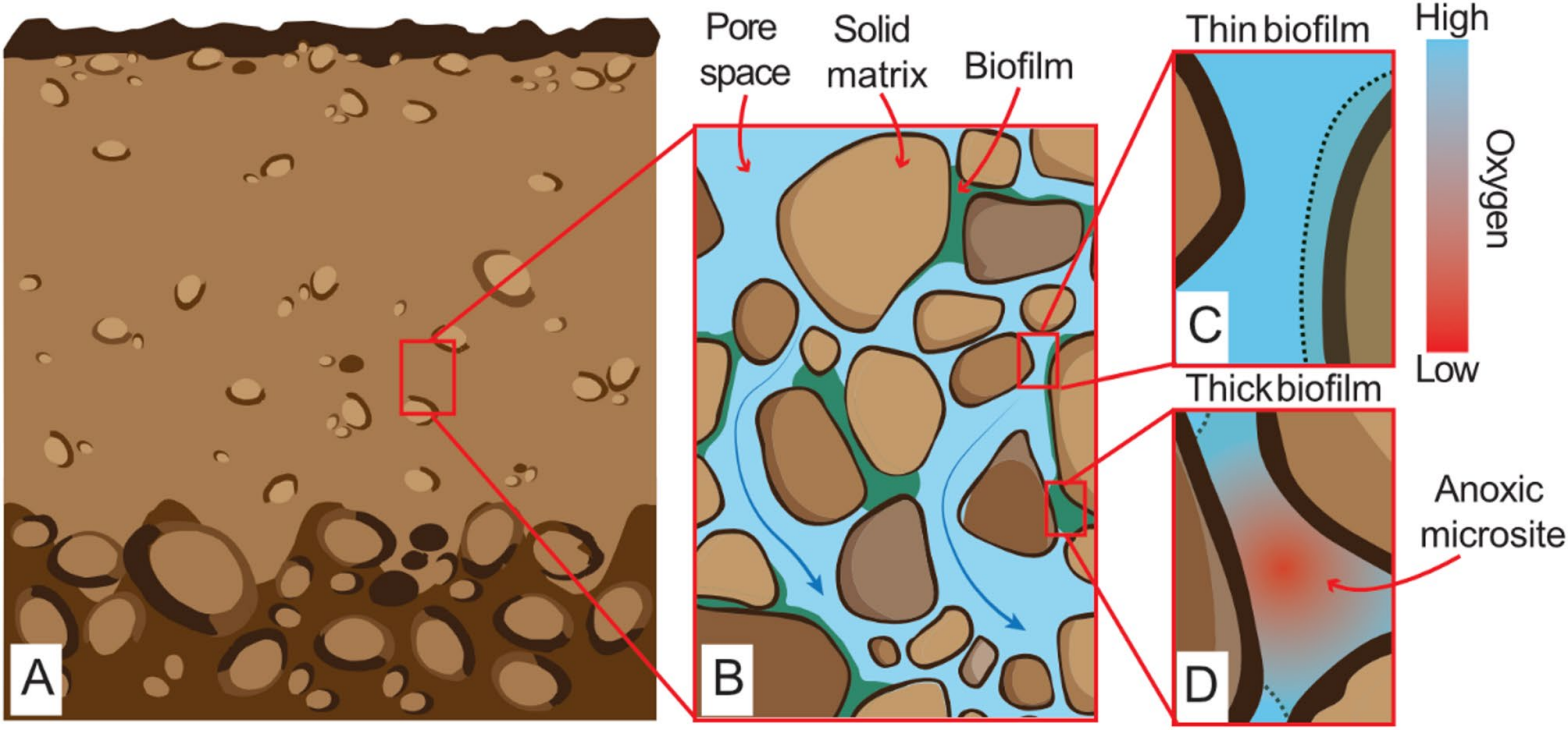
(**a**) Representation of a porous oxic subsurface environment. (**b**) Zoom in of the pore space and solid grains with attached biofilms of variable thicknesses controlled by the solid matrix structure and pore water flow. Depending on the thickness of the biofilm, which mostly controls the microscale balance between O_2_ transport and the intensity of aerobic respiration, the biofilm, whose perimeter is indicated as a green dashed line in panels c and d, may remain permanently oxygenated (**c**) or host the formation of an anoxic microsite (**d**).

As Fe(III) is a less thermodynamically favorable electron acceptor than oxygen (O_2_)^10^, it is generally believed that its microbial reduction is restricted to anoxic or O_2_-limited environments, like deep groundwater systems, flooded soils, ferruginous lakes, and wetlands. Nevertheless, organic matter-stabilized Fe(II) in some oxic subsurface environments has been detected. While abiotic processes, including redox reactions mediated by light or humic substances^1^, and influxes of Fe(II) from deeper anoxic sediment and soil layers may contribute to this Fe(II), microbial reduction of ferrihydrite in the presence of O_2_ has been proposed as an alternative or complementary mechanism^8,11–14^. The occurrence of microbial ferrihydrite reduction in oxic systems is often explained by the presence of O_2_-depleted microscopic zones, referred to as anoxic microsites or microenvironments, that remain undetected in averagely well-oxygenated sediments and soils^11,14,15^. Such anoxic microsites are expected to form where O_2_ diffusion is hindered by the sediment porous structure, heterogeneous pore water flow, and outcompeted by the aerobic growth of thick biofilm layers^12,15–19^, as illustrated in Fig. 1. Though transient and small in scale, these anoxic microsites enable localized ferrihydrite reduction and anaerobic respiration even in well-oxygenated bulk systems^8,13,17,19^.

An alternative explanation for the unexpected presence of Fe(II) in oxic, circumneutral subsurface is the metabolic versatility of certain Fe-reducing microorganisms. As early as 1990, *Shewanella putrefaciens*^20^ was shown to reduce soluble Fe(III) complexes while respiring O_2,_ suggesting a hybrid aerobic and anaerobic lifestyle. Though initially overlooked, this idea resurfaced in 2019 when diverse actinobacteria were found to reduce both soluble and solid Fe(III)^21^ in oxic batch incubations. These studies, however, measured O_2_ only at the bulk scale, leaving unresolved whether Fe(III) reduction occurs in the presence of O_2_ or in undetected anoxic microsites. Recent microfluidic experiments using O_2_ sensors confirmed that the facultative Fe reducer, *Shewanella oneidensis* MR-1 (*S. oneidensis*) can reduce Fe(III) in persistently oxic conditions in the microenvironment surrounding cells^16^. This observation confirms that microbial Fe(III) reduction in oxic sediments and soils is not confined solely to anoxic microsites, as previously assumed. Similar hybrid behavior has been observed for facultative denitrifiers^22^, sulfate reducers^23^, *Escherichia coli*^24^, and, recently, for another facultative iron reducer, *Microbacterium deferre*^25^, suggesting this hybrid metabolism may be more widespread than previously recognized.

Nonetheless, the rates and environmental importance of oxic Fe(III) reduction remain unknown. Scaling the rates of oxic Fe(III) reduction is critical to understanding Fe and contaminant mobility in natural and engineered systems. The quantification of Fe(III) reduction rates under oxic conditions poses methodological challenges because Fe(II), produced by microbial ferrihydrite reduction, can be rapidly re-oxidized and re-precipitated as secondary minerals^3^. As a result, Fe(II) may not accumulate in oxic porewaters, explaining why oxic Fe(III) reduction has so far been difficult to detect and rendering dissolved Fe(II) concentrations a poor indicator of microbial reduction rates in such environments.

In this study, we quantify Fe(III) reduction rates by *S. oneidensis* under oxic and anoxic conditions using a ferrozine-based method that stabilizes Fe(II) and prevents re-oxidation^26,27^. Applying these rates to a representative sediment model, we reveal the overlooked contribution of oxic microbial Fe(III) reduction to Fe(II) mobilization in natural environments.

## Results

### Overall oxic and anoxic Fe(II) production rates in batch experiments

**Fig. 2:**
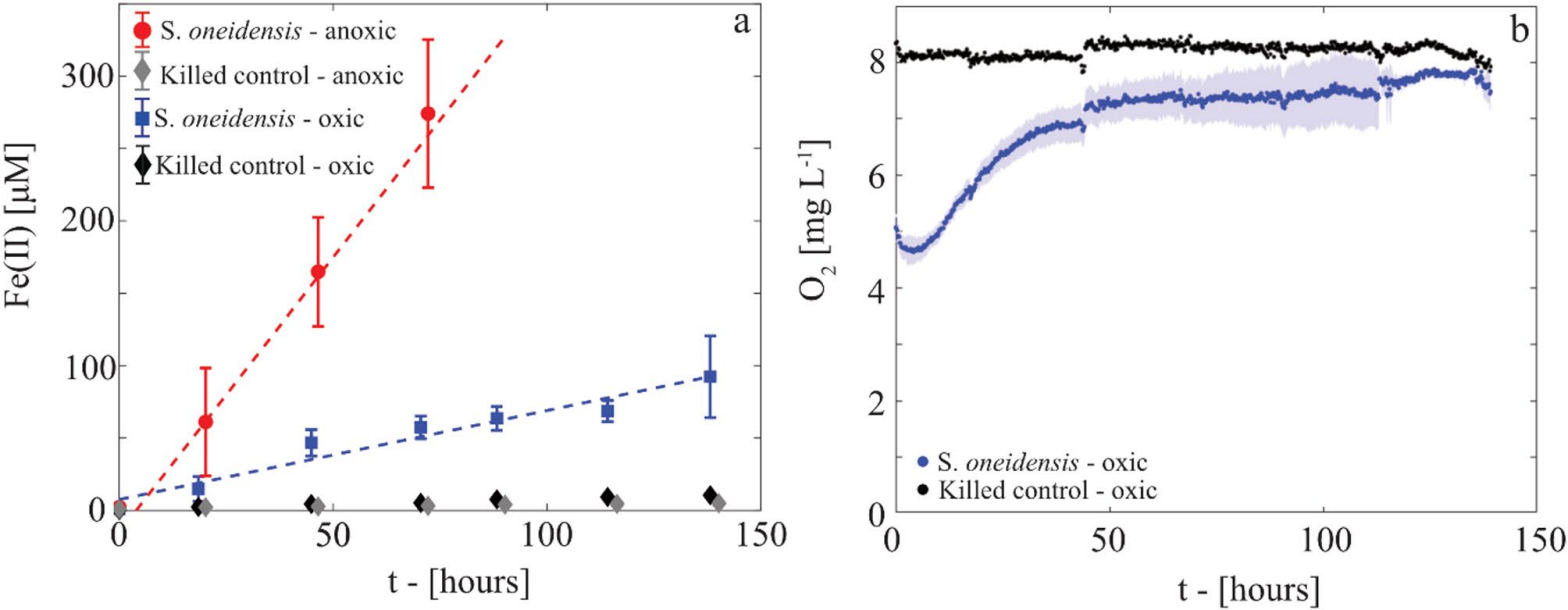
(**a**) Evolution of Fe(II) concentrations in oxic and anoxic *S. oneidensis* incubations and the corresponding killed controls every 24 h for 144 h. A vertical bar indicates the standard deviation computed over triplicates. Results for negative controls are reported in SI (Section S2). Dashed lines indicate the linear interpolation of Fe(II) concentrations. (**b**) Evolution of O_2_ concentrations measured every 10 min by an optical spot sensor in oxic *S. oneidensis* incubations, reported as the average over the 3 replicates. The shaded area indicates the standard deviation around the average. For comparison, O_2_ concentrations measured in one replicate of the killed control are reported (black dots). Time t = 0 h corresponds to ferrihydrite and FZ addition to *S. oneidensis* cultures.

*S. oneidensis* was grown in the dark in 10% (v/v) Luria-Bertani broth, supplemented with 2 mM ferrihydrite—reflecting concentrations typically found in soils^3^—and sufficient (1 mM) ferrozine (FZ) to chelate the expected Fe(II) produced during 144 h without interfering with bacterial respiration^16,20^ (further details in SI, Section S1). In this liquid medium, the bacteria mediate Fe(III) reduction under both oxic and anoxic environments and Fe(II) progressively accumulated as stable FZ-Fe(II) complexes (Fig. 2a)^20,26,27^.

Under anoxic conditions, Fe(II) concentrations (Fig. 2a) exceeded 300 µM after 72 h, indicating efficient ferrihydrite respiration by *S. oneidensis*. These Fe(II) levels surpassed the chelating capacity of 1 mM FZ (Supplementary Information, SI, Section S1 for further details). Fe(II) measurements are therefore reported only for up to 72 h, during which Fe(II) concentrations increased linearly. Under oxic conditions, Fe(II) concentrations also increased linearly, reaching 92.5 ± 28.2 µM at 144 h (Fig. 2a). In other words, ferrihydrite reduction occurred at a constant rate, although slower than under anoxic conditions. Ongoing aerobic respiration by *S. oneidensis* after the addition of ferrihydrite and FZ is evidenced by the lower O_2_ concentrations in live *S. oneidensis* incubations compared to killed controls under the same stirring conditions (Fig. 2b).

The slope of the linear trend was used to quantify the overall Fe(II) production rate for *S. oneidensis* incubations which includes both abiotic and biotic contributions. Killed (Fig. 2a) and negative (SI, Section S2) controls exhibited small increases in soluble Fe(II), ranging from 4 to 6 µM for anoxic conditions and 9–12 µM under oxic conditions after 144 h of incubation. These results suggest that abiotic ferrihydrite reduction, possibly driven by reducing groups contained in organic matter^20,27^, and reactions involving dead cell material (biomass) contributed minimally to the reduction of Fe(III) in Fe-oxides, as observed under both anoxic^20,27^ and oxic conditions^28^. Linear fitting of the controls estimated the mean abiotic Fe(II) production rates to be 0.078 ± 0.0048 µM h⁻¹ (mean ± standard deviation, R² = 0.99) in oxic conditions and 0.034 ± 0.0043 µM h⁻¹ in anoxic conditions. After subtracting the calculated abiotic contribution, the overall Fe(II) production rates attributed to microbial activity were determined to be 0.54 ± 0.089 µM h⁻¹ under oxic (*R*_*O*_) and 3.8 ± 0.43 µM h⁻¹ under anoxic (*R*_*A*_) conditions. Remarkably, the overall oxic Fe(II) mobilization rates were only ~ 7 times lower than those observed in anoxic conditions.

### Oxic and anoxic Fe (III) reduction rate per cell

To normalize Fe(III) reduction rates to possible differences in growth rates under oxic and anoxic conditions, a cell-specific rate was calculated. For this, cell concentrations in batches were estimated in the stationary growth phase, just before the addition of ferrihydrite and FZ.

**Fig. 3:**
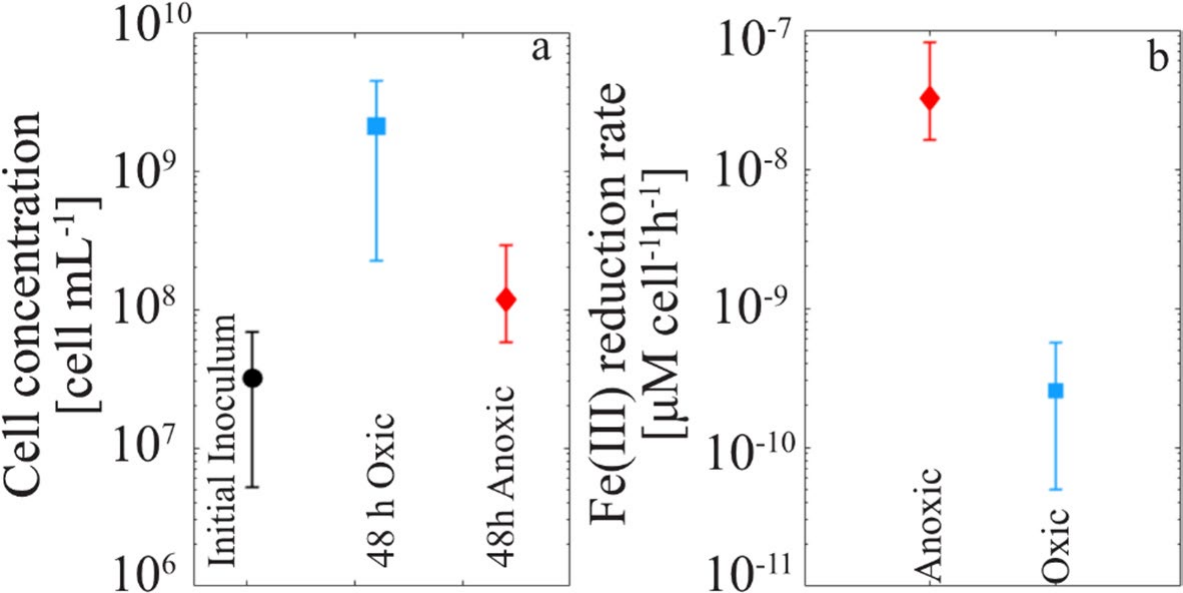
(**a**) Cell concentrations (± standard deviation) of *S. oneidensis* computed for the initial inoculum and after 48 h of incubation under oxic and anoxic conditions. (**b**) Ferrihydrite, Fe(III), reduction rate per cell (± standard deviation) under oxic and anoxic conditions.

The cell concentration increased from the initial inoculum under both anoxic and oxic conditions (Fig. 3a), consistent with protein concentration measurements (Figure S2, SI, Section S3). As expected, bacteria under aerobic conditions grow faster, resulting in cell concentrations one order of magnitude higher (2.1 × 10^9^ ± 2.3 × 10^9^ cell mL^− 1^) than in anoxic conditions (1.2 × 10^8^ ± 5.7 × 10^7^ cell mL^− 1^).

By normalizing the overall Fe(II) production rate in batches computed in previous Section to the cell concentration, we estimated the ferrihydrite reduction rate per cell (Fig. 3b) under oxic (2.6 × 10^− 10^ ± 0.5 × 10^− 10^ µM h^− 1^ cell^− 1^) and anoxic conditions ( 3.2 × 10^− 8^ ± 1.6 × 10^− 8^ µM h^− 1^ cell^− 1^). As a result, cells reduced ferrihydrite ≈ 120 times faster under anoxic than under oxic conditions.

### Modeling of Fe(II) mobilization in oxic sediments

To scale the potential impact of oxic Fe(III) reduction in sediments and soils, information such as mapping of the pore space colonized by biomass and the corresponding microscale distribution of O_2_ concentrations is needed. Methodological limitations and the opacity of soils and sediments prevent direct monitoring in environmental samples^11,19^. Nonetheless, recent advancements in microfluidic approaches have allowed direct measurements of biomass and O_2_ concentration distribution at the pore scale in laboratory analogs, i.e., synthetic and simplified experimental systems that replicate key features of oxic sediments and soils^15,18,22,29,30^. We used a 27 mm-long laboratory analog of sandy sediments presented in the literature^15^ to model microbially-mediated Fe(II) mobilization from ferrihydrite reduction and assess the potential relative contribution of oxic and anoxic ferrihydrite reduction.

**Fig. 4:**
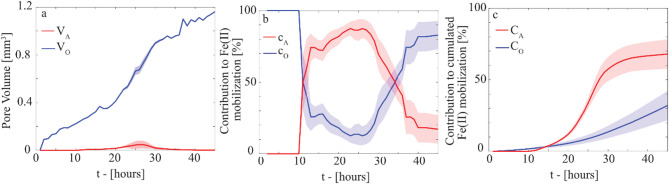
(**a**) Pore space volume occupied by oxic biomass (V_O_) and anoxic microsites (V_A_) as a function of time computed from data in^15^. (**b**) Temporal trend of the relative contribution of oxic biomass (c_O_) and anoxic microsites (c_A_) to the overall Fe(II) mobilization rate during the experiment (Eq. (2), Section “Probabilistic assessment of Fe(II) mobilization in oxic sediments”). (**c**) Cumulative relative contribution of Fe(II) mobilized by anoxic microsites (C_A_) and by oxic biomass (C_O_) during the entire duration of the experiment (45 h), i.e., c_o_ and c_a_ integrated over time for 45 h (Eq. (3), Section “Probabilistic assessment of Fe(II) mobilization in oxic sediments”).

According to the results presented in^15^, the microbial biomass progressively colonized the sediment grain surfaces, eventually occupying up to 1.2 mm^3^ of available pore space at 45 h in the laboratory analog (Fig. 4a), corresponding to 35% of the total pore volume. The system remained oxic at the centimeter scale. Still, anoxic microsites formed transiently, peaking at 26 h with a maximum volume of 0.05 mm^3^ (Fig. 4a), i.e., 1.42% (± 0.99%) of the pore space, and almost disappeared at t > 35 h.

In our model, we assumed a homogeneous distribution of ferrihydrite. Therefore, Fe(II) mobilization was modeled as a ubiquitous process, occurring at every point of the pore space colonized by biomass. However, the rate of Fe reduction varied according to local O_2_ levels, between oxic conditions (*r*_*o*_ = 2.6 × 10^− 10^ µM h^− 1^ cell^− 1^) and anoxic microsites (*r*_*a*_ = 3.2 × 10^− 8^ µM h^− 1^ cell^− 1^, Eqs. (1–3), Section “Fe(III) reduction rates”). The contribution of anoxic microsites to Fe(II) mobilization (*c*_*A*_, Fig. 4b) mirrored their temporal dynamics. Initially, when the system was fully oxic (*t* < 10 h), only oxic Fe reduction occurred in the biomass-colonized pore space. Once anoxic microsites are formed, while occupying only 1–2% of the total pore space, they contribute up to 79–94% to the total Fe(II) mobilization rate. Since oxic Fe(II) mobilization persisted throughout the experiment, the cumulative contribution of oxic biomass (*C*_*O*_, Fig. 4c) accounted for 21%-42% of the total Fe(II) mobilized over 45 h.

## Discussion

Oxic cultures reduced ferrihydrite ~ 7 times more slowly than anoxic cultures (0.54 ± 0.089 vs. 3.8 ± 0.43 µM h⁻¹), yet still mobilized substantial Fe(II). Our observations indicated that Fe(II) concentrations reached 58.6 ± 7.8 µM after 72 h under bulk oxic *S. oneidensis* batch incubations. These results align with previous findings obtained during the growth of *S. oneidensis* on ferrihydrite with 10% v/v LB in an O₂-sensing microfluidic device, where Fe(II) accumulated up to 88.4 ± 19.3 µM over 72 h, while persistent oxic conditions were observed at the microscale^16^. Thus, we rule out the formation of anoxic microsites in our setup and attribute Fe(II) mobilization to oxic, microbially mediated ferrihydrite reduction.

The lower Fe(II) concentrations observed in this study relative to^16^ likely reflect minor alternative complexation, such as those involving organic ligands or soluble FeOH⁺ species. At the neutral pH (~ 7.1) and ferrozine concentration imposed in our study, such complexation may have partially competed with FZ^31,32^, leaving a minor fraction of Fe(II) undetected (see SI, Sect. 4 for further details). In contrast, samples in^16^ were acidified prior to FZ addition, releasing Fe(II) from organic and hydroxyl complexes. Nonetheless, verification by mass spectrometry (SI, Sect. 4) confirmed that our FZ-based approach successfully captured the majority of Fe(II) formed, reliably reflecting the key dynamics of ferrihydrite reduction.

Although FZ–Fe(II) complexation can enhance Fe(III)‑oxide reduction by lowering Fe(II) geochemical activity in solution^26,27^, our oxic 72 h Fe(II) yield matches experiments using similar medium without FZ^16^, indicating no measurable enhancement of Fe(III) reduction in our setup. Therefore, ferrozine proved to be a robust Fe(II) chelating agent in our system. Its successful use in controlled live-cell incubations supports broader application, but extension to natural sediments/soils must be evaluated case by case. Key constraints include pH (reliability likely declines below ~ 4 or above ~ 9), competing ligands, and potential sorption/precipitation effects. A summary of known strengths and limitations is provided in SI, Sect. 4.

Compared to another *Shewanella* strain (*S. putrefaciens*, 12 vs. 240 µM h⁻¹ oxic/anoxic)^20^, *S. oneidensis* exhibits significantly slower Fe(III) reduction rates (~ 100 times) under both oxic and anoxic conditions. This discrepancy can be attributed to several factors. First, differently from Arnold et al.^20^. who used dissolved Fe(III), we fed *S. oneidensis* with solid Fe(III) in ferrihydrite. This form of Fe(III) is more representative of Fe(III) found in natural systems (e.g., rocks^33^, sediments^34^, and soils^7^ ) but likely less bioavailable than the dissolved one. Additionally, strain-specific traits, medium composition and concentration, and incubation temperature may have contributed to the slower Fe(III) reduction rate of *S. oneidensis* compared to *S. putrefaciens*. Indeed, *S. putrefaciens* has one of the highest dissimilatory Fe(III) reduction rates per cell reported in the literature^20^. Moreover, the nutrient conditions and temperature in our study (10% v/v diluted LB broth, equivalent to 2 g L⁻¹ LB; 23 °C) were less favorable than those in *S. putrefaciens* incubations (8 g L⁻¹ Difco broth; 30 °C)^20,35^.

It is worth noting that a minor abiotic contribution to ferrihydrite reduction was observed in both oxic and anoxic control incubations, though it was significantly lower than the microbially mediated reduction. This aligns with previous studies where Fe(III)-oxides (hematite or ferrihydrite) were observed to undergo abiotic reduction under both anoxic^26,27^ and oxic^28^ incubations. This process is attributed to the complex composition of natural organic matter, which contains reduced functional groups capable of acting as electron shuttles for Fe(III) under anoxic conditions while retaining their reducing capacity in the presence of O_2_^28^. In our incubations, the yeast extract, the major constituent of LB broth, was used to mimic the complex composition of natural organic matter^36^, likely contributing to abiotic ferrihydrite reduction.

The Fe(III)-ferrihydrite reduction rate per cell under anoxic conditions (3.2 × 10⁻⁸ ± 1.6 × 10⁻⁸ µM h⁻¹ cell⁻¹ at a cell concentration of 1.2 × 10⁸ ± 5.7 × 10⁷ cells mL⁻¹) aligns with previous findings for the same strain at a similar cell concentration (1.1 × 10⁻⁸ ± 8.1 × 10⁻¹⁰ µM h⁻¹ cell⁻¹ at 6.7 × 10⁸ cells mL⁻¹)^37^, reinforcing the reliability of our results. Interestingly, although the cell-specific Fe(III) reduction rate under oxic conditions is two orders of magnitude lower than under anoxic conditions, the total amount of Fe(II) mobilized in oxic conditions is less than one order of magnitude slower than in anoxic incubations. This discrepancy is due to the significantly higher cell density attained under oxic conditions, emphasizing the need to consider the collective activity of the population rather than just individual cell efficiency in Fe(III) reduction in large-scale environmental systems.

Our study was not designed to elucidate the physiological mechanisms by which *S. oneidensis* mediates Fe(III) reduction in the presence of O₂, which would require a dedicated genetic investigation as done in^25^ for *Mirobacterium deferre*. However, if Fe(III) uptake serves an energetic purpose in parallel to O_2_, as suggested by Arnold et al.^20^ and as shown for *Mirobacterium deferre*^25^, the significantly lower Fe(III) reduction per cell under oxic conditions supports the idea that O₂ remains the preferred electron acceptor for this facultative Fe(III) reducer. This quantitative difference could also result from distinct Fe(III) reduction mechanisms, such as indirect electron shuttling mediated by organic matter and radicals^10^. Additionally, the observed variations in cell shape and size between oxic and anoxic conditions (Figure S3, SI, Section S3) further suggest potential differences in the ecophysiology of *S. oneidensis*.

Although oxic microbially mediated Fe(III)-ferrihydrite reduction occurs ~ 100 times slower than under anoxic conditions, it could play a crucial role in environments where oxic conditions dominate and anoxic microsites constitute a minority of the pore space^12,14^. Our model simulation revealed that oxic ferrihydrite reduction can contribute approximately one-third of Fe(II) mobilized in the lab sediment analog within just a few days. This unexpectedly large contribution is explained by the fact that the cells exposed to oxic conditions constitute the majority of the biomass growing in the pore space of the oxic aquifer lab analog. Despite the slower instantaneous rate, Fe(III) reduction under oxic conditions occurs continually in the background, gradually overtaking an increasing portion of the pore space as biomass colonizes the porous medium. Similar conditions are likely to occur in certain subsurface environments, including well-drained and unsaturated soils, capillary fringes, and shallow aquifers, among the most microbially active environmental systems.

Despite their limited spatial extent and temporal duration, anoxic microsites host most of the Fe(II) mobilization in oxic sediments. Their contribution is inherently dynamic and halts immediately upon microsite dissipation. This transient behavior is expected in natural sediments and soils^14,38^, and is likely driven by poorly constrained factors such as water saturation and nutrient distribution^19^. Our assessment reveals that the main uncertainty in quantifying Fe(II) mobilization rates stems from the lack of high-resolution O₂ measurements. This limitation likely leads to a systematic underestimation of oxic Fe(III) reduction, underscoring the need for improved microscale O_2_ mapping in future studies.

Our approach estimates relative contribution of oxic vs. anoxic reduction, not absolute Fe(II) concentrations. It omits factors like microscale environmental gradients (pH gradients, shear stress, and nutrient availability)^39–41^, potential physiological differences between oxic and anoxic Fe(III) reduction, and Fe(II) reactions in pore spaces, such as complexation, oxidation, or interactions with O_2_ radicals. Despite these simplifications, it provides a useful framework for quantifying Fe(II) mobilization rates and enhancing our understanding of Fe cycling in oxic subsurface environments.

## Conclusions

Our study shows that *S. oneidensis* mediates ferrihydrite reduction under oxic conditions, driving Fe(II) mobilization even in sediments with limited or transient anoxic pore space volume. Anoxic microsites remain the dominant Fe(II) source, but their contribution is short-lived and spatially limited, underscoring the need to account for microscale O₂ gradients and biomass distribution in biogeochemical models.

The impact of different environmental factors (e.g., type and concentration of nutrients and ferrihydrite, pH, temperature, etc.) on oxic Fe(III) reduction rate is yet to be investigated. However, our results highlight the need to integrate ferrihydrite reduction into Fe cycle conceptualizations across oxic subsurface environments, such as shallow aquifers, capillary fringes, soils, and coastal and lake sediments. Even if Fe(II) remains undetected, oxic Fe(III) reduction might still occur, hidden by rapid Fe(II) re-oxidation by O_2_.

Accurate ferrihydrite reduction rates are critical for predicting contaminant and nutrient release. Oxic microbial ferrihydrite reduction may explain elevated arsenate, chromate, and other metal levels in well-oxygenated systems^8,42,43^. Moreover, oxic microbial ferrihydrite reduction may have interesting applications in bioremediation^44^. Fe(III) reducers generate Fe(II)^45^, a strong reductant, capable of transforming pollutants like chlorinated solvents^45,46^ in oxic subsurface environments.

## Methods

### Experimental medium

A liquid medium was prepared with deionized water, 10% v/v Luria Bertani broth (LB, Sigma Aldrich), and 20 mM PIPES (piperazine-N, N′-bis(2-ethanesulfonic) acid, Thermo Scientific). After adjusting the pH to ~ 7.1 using HCl (37%, Sigma Aldrich), the medium was sterilized by autoclaving.

### Incubation setups

**Fig. 5:**
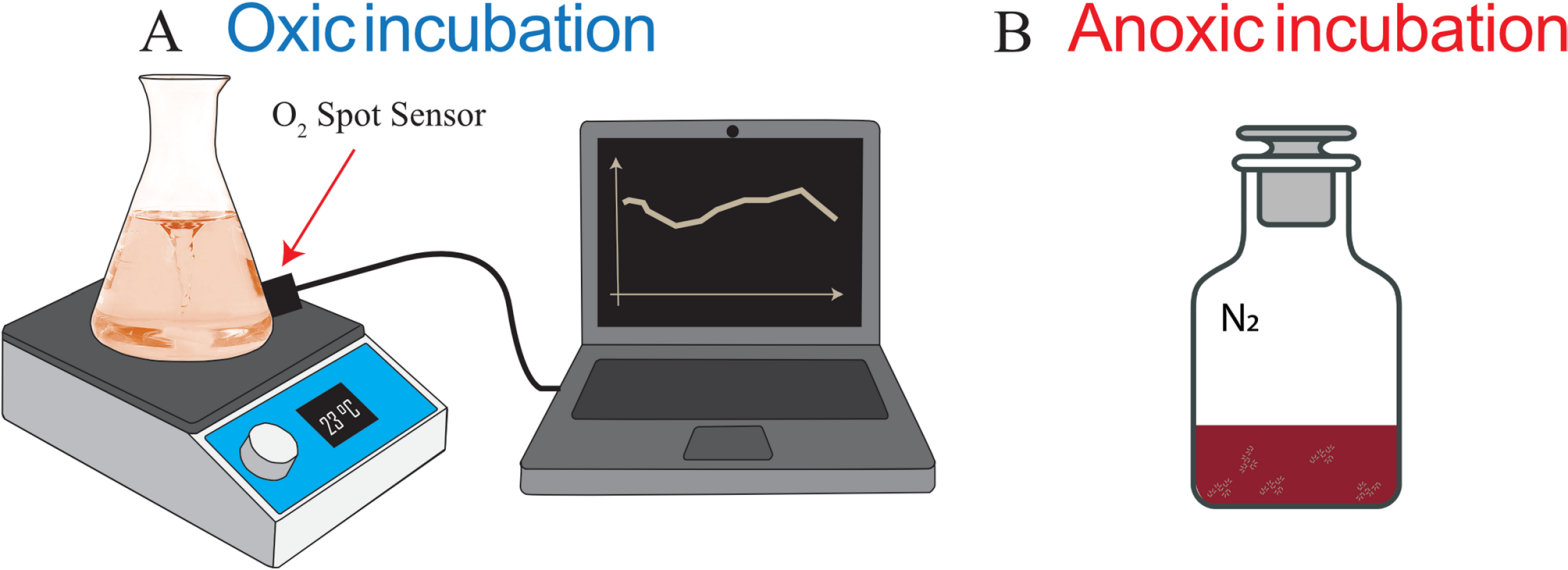
Sketches of *S. oneidensis* incubation setups under oxic conditions(a) and anoxic conditions (b).

The same experimental medium was used in oxic and anoxic incubations. The oxic incubation (Fig. 5A) was performed in a 50 mL sterile Erlenmeyer flask, where a magnetic stirrer continuously stirred 25 mL of medium to maintain air-saturation conditions in the liquid phase. The flask was equipped with a 5 mm spot sensor (OXSP5 supplied by PyroScience) to monitor real-time bulk oxygen (O_2_) concentrations. A sterile porous sponge lid sealed the vial to avoid airborne contamination. For anoxic incubations (Fig. 5B) 25 mL of sterile medium was placed in a 60 mL sterile serum vial crimped with a black butyl rubber lid and purged with 0.22 μm-filtered N_2_. To keep anoxic and oxic setups consistent, stirring was imposed also under anoxic conditions.

Both oxic and anoxic incubations were inoculated with the same aliquot (1:50 v/v) of an aerobic culture of *Shewanella oneidensis* MR-1 (*S. oneidensis*), grown overnight in LB liquid broth at 30 °C and incubated in an orbital shaker (180 rpm). *S. oneidensis* is a facultative Fe reducer commonly used to study Fe(III) reduction under anoxic conditions^47,48^, and it was recently found capable of mediating ferrihydrite reduction under oxic conditions^16^.

After 48 h of inoculation, corresponding to the early stationary phase of *S. oneidensis* growth in both oxic and anoxic incubations (see SI, Section S3), 2 mM of ferrihydrite was added to triplicate cultures. The mineral was synthesized in the laboratory^49^ and always manipulated under the laminar-flow hood. Therefore, it is expected to be practically sterile. However, to prevent any possible contamination from the environment, dry ferrihydrite was UV-treated for 20 min before its addition to the incubations. This ferrihydrite concentration was chosen to reflect typical levels found in soils^3^. Mineral addition in the anoxic incubations was handled in a glove box (N_2_ atmosphere, Jacomex) to avoid O_2_ . *S. oneidensis* was then incubated with ferrihydrite for six days (144 h) under anoxic and oxic conditions. Uninoculated sterile medium and inoculated medium fixed with 4% formaldehyde (each in triplicate) were also incubated as negative and killed controls, respectively, under oxic and anoxic conditions. All the vials were wrapped in aluminum foil to protect them from light.

### Cell count

At the time of inoculation and at 48 h, before adding ferrihydrite to the culture, 1 mL aliquots of each incubation were sampled and fixed with 4% formaldehyde (final concentration). Cells were stained with DAPI (4’,6-diamidino-2-phenylindole, final concentration 1 µg mL^− 1^) and, after 15 min of reaction time, the sample was pipetted into a microfluidic device and imaged using an inverted microscope in the DAPI fluorescence channel. Images were post-processed to count cells and estimate cell concentrations during the stationary phase. Details on the microfluidic cell counting procedure are included in SI, Section S3.

### Temporal dynamics of Fe(II) concentrations

Immediately after adding ferrihydrite, a filtered-sterilized 50 mM Ferrozine (FZ,3-(2-pyridyl)-5,6-diphenyl-1,2,4-triazine-p, p’-disulfonic acid monosodium salt, Sigma Aldrich) stock solution was added to the incubations in a final concentration of 1 mM. Ferrozine is a well-known Fe(II) chelator that forms a magenta complex with Fe(II), which has an absorbance peak at 560 nm. The complex traps the Fe(II) potentially produced by microbially-mediated Fe(III) reduction^20,26,27,32^, preventing its re-oxidation or re-precipitation into secondary mineral phases.

Fe(II) concentration was monitored every 24 h in each vial by measuring the absorbance of the FZ-F(II) complex via a spectrophotometric method (see SI, Section S1 for details). At the end of the incubations, the measured Fe(II) concentrations in the oxic incubations were verified by mass spectrometry (see SI, Section S4 for details and results).

### Fe(III) reduction rates

The temporal evolution of Fe(II) concentration was linearly fitted using *cftool* MATLAB^®^ (R2021b, version 9.11.0.1769968) to estimate the overall Fe(III) reduction rates under oxic (*R*_*O*_, [µM h^− 1^]) and anoxic (*R*_*A*_, [µM h^− 1^]) conditions. Microbial growth attained the stationary phase at t < 48 h. Therefore, the number of alive cells is constant. We normalized the overall Fe(III) reduction rates to the cell concentrations at 48 h to determine the Fe(III) reduction rate per cell under oxic (*r*_*o*_, [µM h^− 1^ cell^− 1^]) and anoxic (*r*_*A*_, [µM h^− 1^ cell^− 1^]) conditions.

### Probabilistic assessment of Fe(II) mobilization in oxic sediments

Estimating the relative contribution of oxic and anoxic ferrihydrite reduction in oxic sediments to Fe(II) mobilization requires mapping the pore space colonized by biomass and the corresponding microscale distribution of O_2_ concentrations. To date, such pieces of information are not accessible in real sediments^11,19^. However, direct measurements of biomass and O_2_ concentration distribution at the pore scale have been recently collected using novel microfluidic approaches simulating oxic sediments and soils^15,18,22,29,30^ that we used here as a reference to perform our probabilistic assessment.

We used the data presented in^15^ that simulated the porewater flow in sandy sediment progressively colonized by an aerobic model strain *Pseudomonas putida* GB1. Biomass and O_2_ concentrations were mapped at the microscale hourly for 45 h to identify the portion of pore space colonized by biomass and characterized by anoxic conditions, i.e., anoxic microsite volume. To the best of our knowledge, this is the only study where O_2_ and biomass were mapped simultaneously in a heterogeneous confined environment, and no similar studies are available for *S. oneidensis* to date. By elaborating on this dataset, we tracked the time evolution of pore space volume occupied by oxic biomass (*V*_*O*_) and anoxic microsites (*V*_*A*_) (Fig. 4a, see SI Section S5, for computational details).

We assumed that ferrihydrite was homogeneously distributed in the pore space and that all the cells growing in the sediment could perform ferrihydrite reduction, switching between oxic (*r*_*O*_) and anoxic (*r*_*A*_) rates as a function of the microenvironment experienced by the cells. In other words, ferrihydrite reduction is feasible in every portion of the pore space colonized by biomass independently of the redox state, with rates varying in space and time, responding to the local O_2_ concentration.

The total Fe(II) mobilization rate (*M*_*T*_ [µM h^− 1^]) resulted from the sum of oxic (*M*_*O*_) and anoxic biomass (*M*_*A*_) contributions expressed as follows

1 $$\:\left\{\begin{array}{c}{M}_{A}\left(t\right)={r}_{A}\:{\rho\:}_{cell}{\:V}_{A}\left(t\right)\\\:\:\:{M}_{o}\left(t\right)={r}_{o}{\:\rho\:}_{cell}{\:V}_{o}\left(t\right)\:\:\:\end{array}\right.$$

Here, *r*_*A*_ and *r*_*O*_ [µM cell^− 1^ h^− 1^] are the ferrihydrite reduction rates estimated from anoxic and oxic *S. oneidensis* incubations and cell counting, while *ρ*_*cell*_ [cell mm^− 3^] is the cell density per unit of pore space. The quantities *V*_*A*_ and *V*_*O*_ are the pore space volume [mm^3^] occupied by anoxic microsites and oxic biomass in the oxic sediment elaborated from^15^. (Fig. 4A).

To assess the instantaneous relative contribution of anoxic microsites (*c*_*A*_) and oxic biomass (*c*_*O*_) to Fe(II) mobilization rate, we assumed a constant and uniform cell density in the pore space occupied by biomass, and Eq. (1) is recast into

2 $$\:\left\{\begin{array}{c}{c}_{A}\left(t\right)=\frac{{M}_{A}}{{M}_{T}}=\frac{{r}_{A}{\:V}_{A}\left(t\right)}{{r}_{A}{\:V}_{A}\left(t\right){+r}_{o}{\:V}_{o}\left(t\right)}\\\:\:\:{c}_{o}\left(t\right)=\frac{{M}_{O}}{{M}_{T}}=\frac{{r}_{O}{\:V}_{O}\left(t\right)}{{r}_{A}{\:V}_{A}\left(t\right){+r}_{o}{\:V}_{o}\left(t\right)}\:\:\:\end{array}\right.$$

 

To assess the relative impact of oxic biomass (*C*_*O*_) and anoxic microsite (*C*_*A*_) in Fe mobilization on a longer timescale, the relative contribution of Fe(II) mobilized by anoxic microsites and oxic biomass was then compared to Fe(II) mobilized cumulated over 45 h, i.e.,

3 $$\:\left\{\begin{array}{c}{C}_{A}\left(t\right)=\frac{\sum\:_{0}^{t}{r}_{A}{\:V}_{A}\left(t\right)}{\sum\:_{0}^{T}\left[{r}_{A}{\:V}_{A}\left(t\right){+r}_{o}{\:V}_{o}\left(t\right)\right]}\\\:\:\:{C}_{o}\left(t\right)=\frac{\sum\:_{0}^{t}{r}_{O}{\:V}_{O}\left(t\right)}{\sum\:_{0}^{T}\left[{r}_{A}{\:V}_{A}\left(t\right){+r}_{o}{\:V}_{o}\left(t\right)\right]}\:\:\:\end{array}\right.$$

 

Here, *t* refers to the time at which C_A_ and C_O_ are evaluated, while *T* is the total duration of the experiment. i.e., 45 h in this case. The uncertainty associated with the input parameters (*r*_*O*_ and *r*_*A*_) and predictors (*V*_*O*_ and *V*_*A*_) is incorporated into the analysis and propagated to the final outputs using a Monte Carlo approach (see details in SI, Section S5).

## Supplementary Information

Below is the link to the electronic supplementary material.

Supplementary Material 1

## Data Availability

The original contributions presented in the study are included in the article/Supplementary material, further inquiries can be directed to the corresponding author.
