# Supplementary material for: Oxic microbial ferrihydrite reduction rates of Shewanella oneidensis and the potential for Fe mobilization in oxic sediments
Source: Sci Rep. 2025 Aug 26;15:31343. doi: 10.1038/s41598-025-16963-w (PMC12381104; doi:10.1038/s41598-025-16963-w)
Supplement: Supplementary file 1 — Supplementary Material 1 [file 41598_2025_16963_MOESM1_ESM.docx]

**Supplementary Information: “Oxic microbial ferrihydrite reduction rates of *Shewanella oneidensis* and the potential for Fe mobilization in oxic sediments”**

Giulia Ceriotti^1*^, Alice Bosco-Santos^2^, Jasmine S. Berg^1^

^1^Institute of Earth Surface Dynamics, Faculty of Geoscience and Environment, University of Lausanne, Lausanne, Switzerland

^2^ Institute of Earth Sciences, Faculty of Geoscience and Environment, University of Lausanne, Lausanne, Switzerland

E-mail: [giulia.ceriotti@unil.ch](mailto:giulia.ceriotti@unil.ch)

# Measurements of Fe(II) concentrations

The addition of 1 mM of Ferrozine (FZ) rapidly creates a stable complex FZ-Fe(II) in case Fe(II) was released by biotic or abiotic processes in our incubations.

Concerning ferrozine concentration, 1mM was chosen to minimize ferrozine concentration as much as possible, to reduce the risk of artificially enhancing ferrihydrite reduction [1, 2]. At the same time, ferrozine concentration should be high enough to fully capture the dynamics of oxic ferrihydrite reduction over 144 hours. Based on [3] that found that *S. oneidensis* increased the Fe(II) concentration of 100-115 μM in 72 hours, it is reasonable to accumulate a Fe(II) concentration between 200-230 μM in 144 hours. Such Fe(II) concentration would require 600-660 μM of ferrozine to be chelated. To avoid the potential slowdown of the chelating process due to low ferrozine concentration, the ferrozine concentration was increased to a final concentration 1 mM.

Arnold et al. (1990) showed that a concentration of ferrozine of ~3 mM did not interfere with *S. putrefaciens* growth and oxygen respiration rate. Being the selected concentration of 1 mM, three times smaller than the one tested by Arnold et al. (1990), we can confidently exclude its interference with S. oneidensis respiration rates.

The Fe(II)-FZ complex concentration can be measured by spectrophotometric methods under both anoxic and oxic conditions [1, 2, 4].

To this end, we applied the following protocol:

- A sample of 300 μL was collected from the incubations every 24 hours with sterile material, i.e., with a pipette under laminar flow or with a syringe flushed three times with filter-sterile N_2_ for oxic and anoxic batches, respectively.
- The sample was transferred to a 1.5 mL Eppendorf centrifuge conical tube and centrifuged at 3000 x g for 5 minutes to precipitate suspended ferrihydrite particles.
- An aliquot of 200 μL of the supernatant was pipetted into a Greiner Microplate micro-well (96 wells, PS, F-bottom, clear), and the sample’s absorbance at 560 nm was measured using a Spark® Multimode Microplate Reader.

Such a protocol is a modified version of the one originally proposed by Arnold et al. (1990)[4]. Specifically, the modifications consisted in:

- The concentration of ferrozine introduced in the culture was reduced from 3 mM to 1mM. This concentration was sufficient to capture the expected dynamics of oxic ferrihydrite reduction based on the observations presented in a previous study[3].
- The sampling interval was longer compared to Arnold et al. who employed high-resolution sampling on the order of seconds to capture rapid changes. This study focuses on longer-term dynamics at the day-to-week scale. Therefore, a 24-hour sampling interval was sufficient for this purpose.
- Arnold et al. preserved samples by freezing them until spectrophotometric analysis. In this study, measurements were conducted immediately after sampling.

The absorbance measurements are translated into Fe(II) concentrations by applying the following calibration curve

$$\left\{ \begin{aligned} Fe\left( II \right)\left[ \mu M \right]=167.52ABS-7.70 if ABS\leq0.34 \\ Fe\left( II \right)\left[ \mu M \right]=69.51ABS+25.70 if ABS>0.34 \end{aligned} \right.$$

The calibration curve was obtained by interpolating the absorbance measured for 4 calibration solutions with Fe(II) concentrations equal to 0, 50, 100, and 200 μM. To prepare the calibration solutions, a stock solution of 2 mM of Fe(II) was prepared by dissolving Fe-sulfate in the sterile anoxic experimental medium (10% v/v LB, deionized water, 20 mM PIPES). The stock solution is diluted into the sterile oxic medium previously enriched with 1 mM FZ to obtain the four calibration solutions. By adding the anoxic stock solution to the medium already enriched by 1 mM FZ, the Fe(II) was fast trapped by FZ, preventing Fe(II) oxidation by O_2_. Since the Fe(II) is trapped by FZ, the same calibration curve is applied to oxic and anoxic incubations. The Fe(II)-FZ complex exists in a molar ratio of 1:3[5]. This means that 1 mM of FZ is sufficient to trap a maximum of ~300μM of Fe(II).

# Negative controls

Fe(II) concentration measurements for *S. oneidensis* incubations and killed controls under oxic and anoxic conditions presented in the manuscript are here compared against the negative controls (Figure S 1). Negative and killed control results are consistent under both anoxic and oxic conditions, confirming the insignificance of abiotic ferrihydrite reduction and FZ alteration compared to microbially-mediated processes, as further discussed in the manuscript.


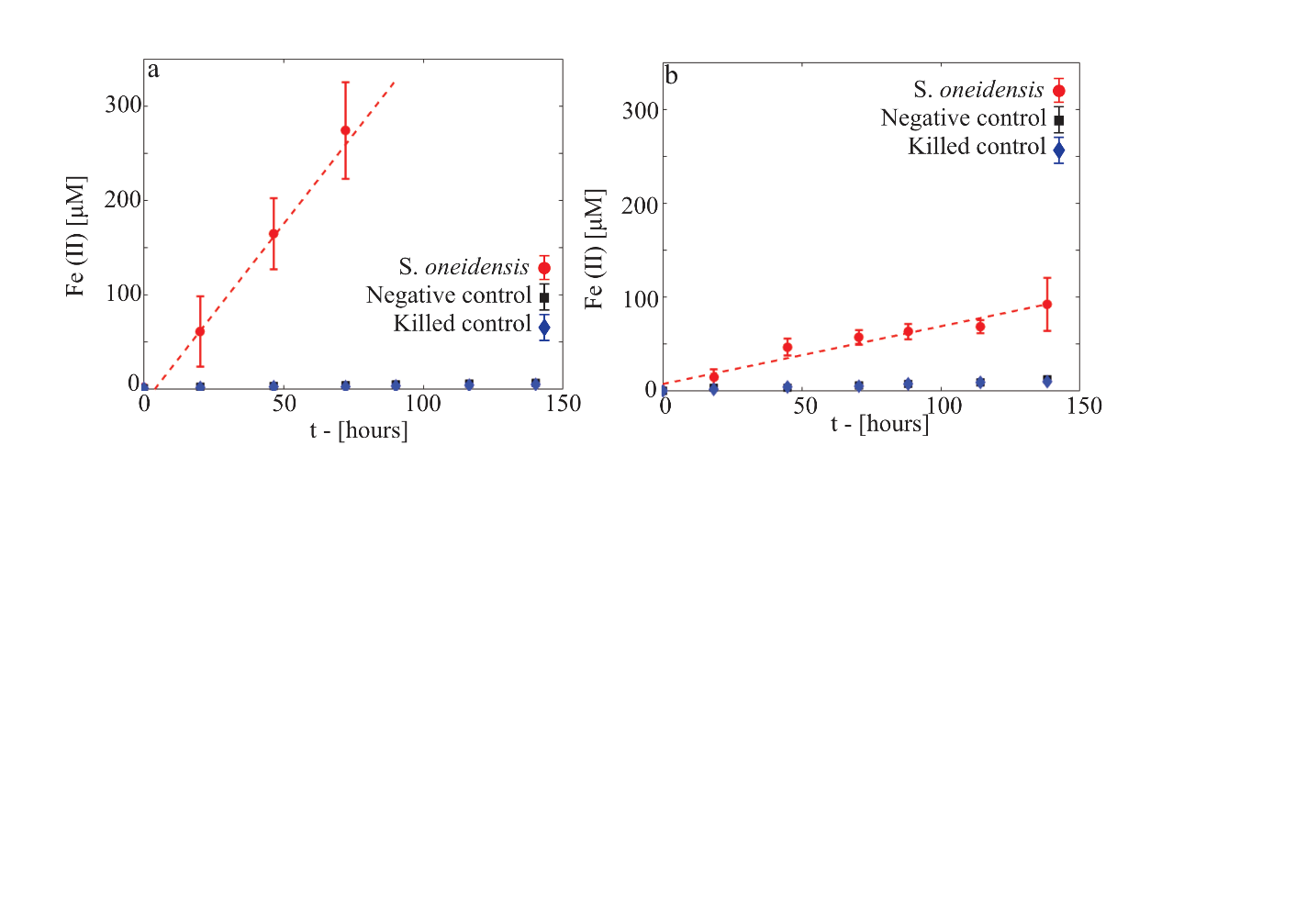


Figure S 1 - Time evolution of Fe(II) concentration in S. oneidensis incubations, killed and negative controls measured every 24 hours for 144 hours under anoxic (a) and oxic (b) conditions. A vertical bar indicates the standard deviation computed over triplicate for each experiment. Dashed lines indicate the linear interpolation of -Fe(II) concentration data.

# Identification of the stationary growth phase and cell counting procedure

We characterized the temporal dynamics of *S. oneidensis* growth under oxic and anoxic conditions (3 replicates for each condition, Figure S 2) in the chosen experimental medium, i.e., 10% v/v LB, deionized water, 20 mM PIPES, and 2 mM ferrihydrite. For experimental setups and inoculation procedures, the reader can refer to the Methods presented in the manuscript. No FZ was added to these incubations to avoid interference with the biomass content spectrophotometric measurements.

We monitored bacterial growth for 168 hours (7 days) every 24 hours using protein concentration as a proxy and the following procedure:

- A sample of 300 μL was collected from the incubations with sterile material (with a pipette under laminar flow or with a syringe flushed 3 times with filter-sterile N_2_ for oxic and anoxic batches, respectively).
- The sample was transferred in a 1.5 mL Eppendorf centrifuge conical tube and mixed with 345 μL of 0.1 M HCl for 15 minutes. This step lysed bacterial cells and freed their protein content.
- An aliquot of 150 μL was pipetted into a micro-well of a Greiner Microplate (96 wells, PS, F-bottom, clear) and mixed with 150 μL of Coomassie brilliant blue G-250.
- After 10 minutes of contact time, the sample’s absorbance at 595 nm (ABS595) was measured using a Spark® Multimode Microplate Reader.
- Protein concentration was computed through the following calibration curve obtained by applying Thermo Scientific's guidelines.

$$Protein concentration \left[ \frac{\mu g}{mL} \right]=68.7 {ABS}_{595}-18.1$$

Oxic *S. oneidensis* growth attained the stationary phase after 24 hours, while, under anoxic conditions, protein concentration can be considered stable only after 48 hours. This is because of an initial lag phase, probably due to the sudden change of oxygenation conditions for the cell inoculum and the need for adaptation. The biomass during the stationary phase under oxic conditions is more abundant than under anoxic conditions, reflecting a more efficient metabolic activity in the presence of oxygen. This means that, given the same medium, imposing anoxic conditions led to a lower biomass density. We concluded that at 48 hours from the inoculation *S. oneidensis* growth attained the stationary phase under both anoxic and oxic conditions.


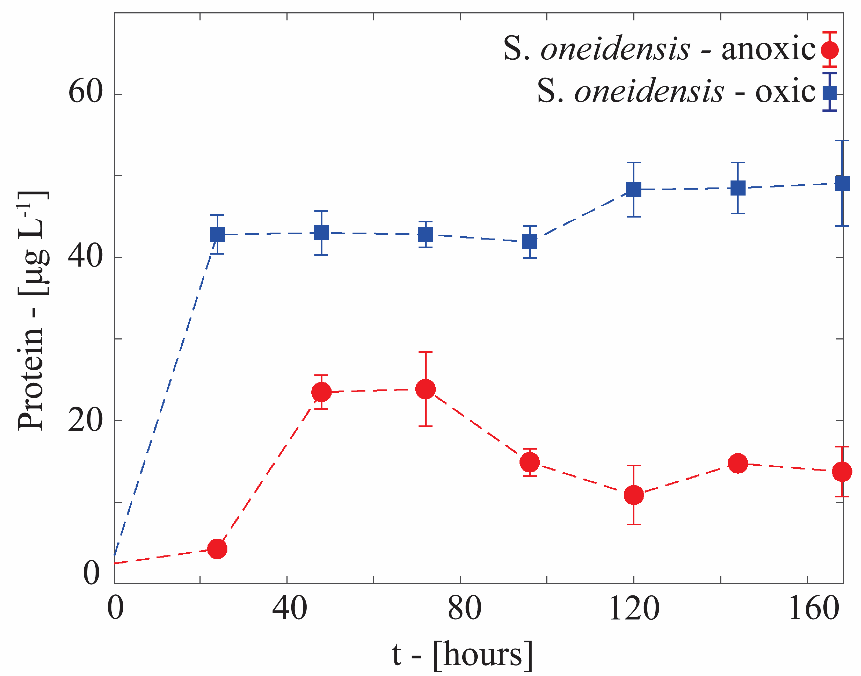


Figure S 2 – S. oneidensis growth assessment in 10 % v/v LB buffered (20 mM PIPES) medium under oxic and anoxic conditions using protein concentration as a proxy.

Cell concentrations were estimated using a cell counting procedure in a 1-mL aliquot sampled at 48 hours from inoculation before adding ferrihydrite and FZ, and fixed with 4% formaldehyde. DAPI was then added to a final concentration of 1 μg mL^-1^. After 10 minutes of reaction time, the samples were injected into a microfluidic device with geometry reported in Figure S 3a. Samples from oxic incubation were diluted 20X to reduce cell density and facilitate the cell counting procedure. The microfluidic device was produced by casting polydimethylsiloxane polymer structure (PDMS, Sylgard 184 Silicone Elastomer mixed with 10 w/w % of curing agent; supplier: Dow Corning, Midland, MI) using a silica mold prepared with classical soft lithography. The microfluidic device, saturated with a DAPI-stained sample, was then placed on the stage of a Zeiss AxioObserver Z1 inverted microscope equipped with a Photometrics CoolSnap HQ2 camera. After 1 hour of waiting time to allow cell deposition at the bottom of the microfluidic device, DAPI-stained cells were imaged at 10 randomly chosen locations along the microfluidic channel at 20X magnification (EC Plan Neofluar 20X, N.A. 0.5 Ph2) in DAPI fluorescence mode. Images were post-processed to identify cells using an image thresholding procedure (e.g., in Figure S 3b-g) using an in-house-developed MATLAB® code (R2021b, version 9.11.0.1769968).


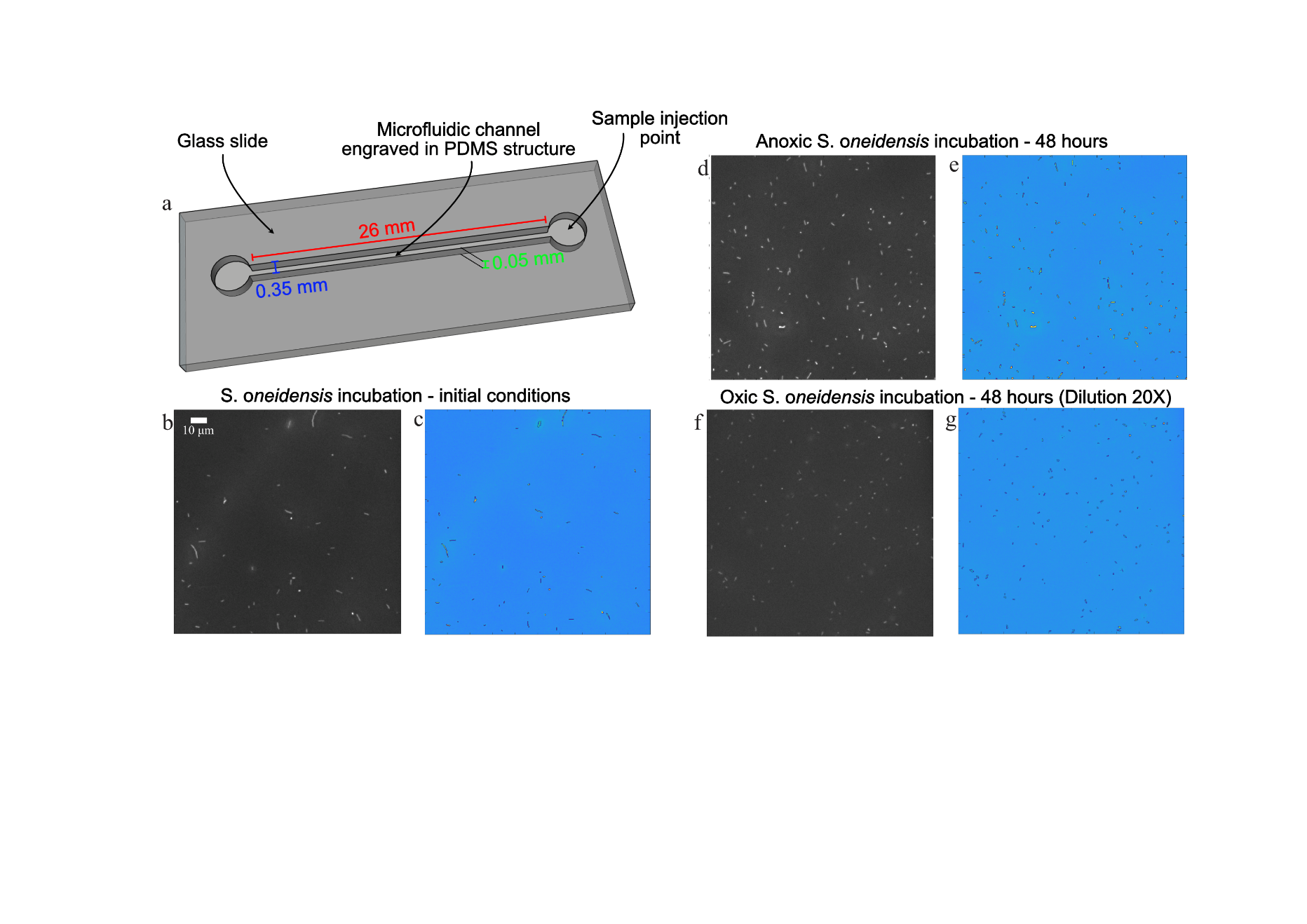


Figure S 3 – a) Outline of the microfluidic device used for cell counting procedure. Portions of raw images captured by the inverted microscope for the initial inoculum (b) and after 48 hours of incubations under anoxic (d) and oxic conditions (f). Panels c, e, and g display processed images corresponding to panels b, c, and f, respectively, with identified cells in yellow color on a light-blue background and delimited by a black perimeter.

The number of cells in every image is normalized to the corresponding imaged sample volume. The cell concentration and its uncertainty (Figure 3a, main text) were estimated by computing the mean and standard deviation from all images taken for each sample.

# Mass spectrometry

At the end of the planned incubation period (i.e., 6 days), the medium was filtered through 0.22 μm syringe filters and stored frozen at −15 °C until further processing. To quantify Fe(II) via mass spectrometry, the Fe(II)–ferrozine (FZ) complex was acid-digested to eliminate matrix interference. For acid digestion, 1 ml of each Fe(II)–FZ complex sample was transferred to a Teflon vial and mixed with 5 ml of metal-free, double-distilled HNO₃ and 0.5 ml of metal-clean H₂O₂. The mixture was heated on a hot plate at 150 °C until complete evaporation to dryness. The resulting residue was then reconstituted in 10 ml of 0.05 M HNO₃. Metal concentrations were measured using an Agilent 8700 triple quadrupole ICP-MS. Fe(II) concentrations determined by mass spectrometry were quantitatively consistent with those obtained via photometric measurements of Fe(II)–FZ complex absorbance. This cross-validation (Table Table S 1) supports the reliability of using Fe(II)–FZ absorbance to monitor Fe(II) production during live bacterial incubations under oxic conditions and confirms the robustness of the results presented in this manuscript.

The reliability of ferrozine to follow Fe(II) dynamics in live cell incubations is also supported by previous studies using a similar setup but with different model strains (*Shewanella Putrefaciens* CN32 and S. putrefaciens sp. strain 200) and organic matter substrates (e.g., extracted from terrestrial and aquatic natural samples). Ferrozine was proven not to interfere with oxygen respiration rates and not to be degraded or altered by microbial activity.[1, 4] These observed properties of ferrozine under controlled experimental conditions make the method used in this study a promising approach to characterizing Fe(II) biogeochemical dynamics, also in natural samples. Some research effort has already been undertaken to assess the performance of ferrozine in combination with more heterogeneous natural samples (e.g., [6]). However, we note that the complexity and heterogeneity of natural sediments and soils present important challenges for the broader application of our Fe(II)-stabilizing method, which have to be carefully assessed on a case-by-case basis. For example, specific properties of each natural sample could influence Fe(II) stability, e.g., mineral or microbial composition that modifies ferrozine stability. A parameter with demonstrated impact on ferrozine stability is pH [5]. We can anticipate that the ferrozine method's reliability in soils and sediments with pH lower than 4 (e.g., affected mining drainage) or above 9 (e.g., calcareous samples) might be limited.

Beyond stability, high pH might also interfere with ferrozine-Fe(II) complex formation itself. Indeed, with increasing pH, the free Fe^2+^ tends to react more and more with OH^-^ to form FeOH^+^. The formation of FeOH^+^ is a very fast process, and ferrozine, especially at low concentrations [7], might not efficiently trap Fe(II) before it reacts with OH^-^. According to the log-species diagram [5], the order of magnitude of FeOH^+^ concentrations compares to those of Fe^2+^ from pH between 8 and 9, possibly largely affecting the ferrozine assay. In this study, pH is imposed at 7.1-7.2 and maintained stable by the buffering agent PIPES. At this pH, the formation of FeOH^+^ cannot be excluded, but we expect it to be minimal, with concentrations more than two orders of magnitude smaller than those of Fe^2+^. This quantitative assessment was confirmed by simulation of Fe(II) speciation at pH = 7.2 in water using the geochemical software PHREEQC, imposing Fe(II) similar to those observed in our batch incubations.

Table S 1 – Comparison of Fe(II) concentration estimated with the ferrozine method and measured via mass spectrometry at the end of oxic incubations (6 days or 144 hours after the addition of ferrihydrite). Concentrations are presented as mean values over the 3 incubation replicates along with their standard deviation for oxic S. Oneidensis incubations, killed, and negative controls.

| **Experiment** | **Mass Spectrometry [μM]** | **Ferrozine [μM]** |
| --- | --- | --- |
| *S. oneidensis* incubation | 112.1 ± 55.4 | 92.5 ± 28.2 |
| Killed control | 10.5 ± 3.6 | 10.5 ± 1.0 |
| Negative control | 12.6 ± 1.2 | 12.9 ± 0.7 |

# Oxic and anoxic biomass

We moved from the data presented in Figure 3D in Ceriotti et al. (2022)[8], where the authors presented the percentage of total pore space colonized by biomass (*PS_B_*) and occupied by anoxic microsites (*PS_A_*) as a function of time. The latter is used here to compute the temporal dynamics of the pore space anoxic volume (*V_A_*) and the associated uncertainty reported in Figure 4A of the manuscript.

The mean trend of *V_O_*, i.e., the volume of pore space colonized by biomass and exposed to oxic conditions, was computed as the difference between the total pore space occupied by biomass (from *PS_B_*, in Ceriotti et al. (2022)[8]) and *V_A_*. The uncertainty associated with anoxic microsite volume was propagated to *V_O_*. The uncertainty associated with *PS_B_* was not accounted for in this work.

# Uncertainty propagation

All the parameters and predictors in Eq. (1) of the manuscript were associated with a certain level of uncertainty that is propagated to the final assessment outputs, namely *c_A_*, *c_B_*, *C_A_*, and *C_B_* (Figure 4b and c). To propagate the uncertainty, we used a Monte Carlo approach with 100 000 realizations. All parameter and predictor values were randomly sampled for each realization, assuming a uniform distribution within their uncertainty intervals.

# References

1. Royer, R.A., et al., *Enhancement of biological reduction of hematite by electron shuttling and Fe (II) complexation.* Environmental science & technology, 2002. **36**(9): p. 1939-1946.

2. Royer, R.A., et al., *Enhancement of hematite bioreduction by natural organic matter.* Environmental science & technology, 2002. **36**(13): p. 2897-2904.

3. Ceriotti, G., et al., *Decoupling microbial iron reduction from anoxic microsite formation in oxic sediments: a microscale investigation through microfluidic models.* Frontiers in Microbiology, 2025. **16**: p. 1504111.

4. Arnold, R.G., et al., *Regulation of dissimilatory Fe (III) reduction activity in Shewanella putrefaciens.* Applied and Environmental Microbiology, 1990. **56**(9): p. 2811-2817.

5. Smith, G.L., et al., *Complexation of ferrous ions by ferrozine, 2, 2′-bipyridine and 1, 10-phenanthroline: Implication for the quantification of iron in biological systems.* Journal of Inorganic Biochemistry, 2021. **220**: p. 111460.

6. Huang, W. and S.J. Hall, *Optimized high-throughput methods for quantifying iron biogeochemical dynamics in soil.* Geoderma, 2017. **306**: p. 67-72.

7. Morgan, B. and O. Lahav, *The effect of pH on the kinetics of spontaneous Fe (II) oxidation by O2 in aqueous solution–basic principles and a simple heuristic description.* Chemosphere, 2007. **68**(11): p. 2080-2084.

8. Ceriotti, G., et al., *Morphology and size of bacterial colonies control anoxic microenvironment formation in porous media.* Environmental Science & Technology, 2022. **56**(23): p. 17471-17480.
